# Commonality and variance of resting-state networks in common marmoset brains

**DOI:** 10.1038/s41598-024-58799-w

**Published:** 2024-04-09

**Authors:** Kanako Muta, Yawara Haga, Junichi Hata, Takaaki Kaneko, Kei Hagiya, Yuji Komaki, Fumiko Seki, Daisuke Yoshimaru, Ken Nakae, Alexander Woodward, Rui Gong, Noriyuki Kishi, Hideyuki Okano

**Affiliations:** 1 Graduate School of Human Health Sciences, Tokyo Metropolitan University, Tokyo, Japan; 2 Laboratory for Marmoset Neural Architecture, Center for Brain Science, RIKEN, Saitama, Japan; 3 Live Animal Imaging Center, Central Institute for Experimental Animals, Kanagawa, Japan; 4 Department of Physiology, Keio University School of Medicine, Tokyo, Japan; 5 Division of Regenerative Medicine, The Jikei University School of Medicine, Tokyo, Japan; 6 Division of Behavioral Development, Department of System Neuroscience, National Institute for Physiological Science, Aichi, Japan; 7 Exploratory Research Center on Life and Living Systems, National Institutes of Natural Sciences, Aichi, Japan; 8 Connectome Analysis Unit, Center for Brain Science, RIKEN, Saitama, Japan

**Keywords:** Neuroscience, Functional magnetic resonance imaging

## Abstract

Animal models of brain function are critical for the study of human diseases and development of effective interventions. Resting-state network (RSN) analysis is a powerful tool for evaluating brain function and performing comparisons across animal species. Several studies have reported RSNs in the common marmoset (*Callithrix* *jacchus*; marmoset), a non-human primate. However, it is necessary to identify RSNs and evaluate commonality and inter-individual variance through analyses using a larger amount of data. In this study, we present marmoset RSNs detected using > 100,000 time-course image volumes of resting-state functional magnetic resonance imaging data with careful preprocessing. In addition, we extracted brain regions involved in the composition of these RSNs to understand the differences between humans and marmosets. We detected 16 RSNs in major marmosets, three of which were novel networks that have not been previously reported in marmosets. Since these RSNs possess the potential for use in the functional evaluation of neurodegenerative diseases, the data in this study will significantly contribute to the understanding of the functional effects of neurodegenerative diseases.

## Introduction

The common marmoset (*Callithrix* *jacchus*; marmoset) is recognized as a promising laboratory animal because it is the only primate that has been genetically modified^1–4^. Considering these advantages, the common marmoset was chosen as the main research animal model in Brain/MINDS, a Japanese brain science research project^5,6^ that aims to evaluate the pathology of neurodegenerative diseases. Species differences are a barrier to transitioning from preclinical studies in rodents to clinical studies in humans, and good performance in rodents often does not translate well into clinical practice. Therefore, translational research using nonhuman primates is extremely important for extrapolating rodent data to humans.

The greatest advantage of genetically modified animal models of congenital diseases is the ability to follow the time course of a disease from the unaffected stage to the onset and terminal stages and to obtain biological samples at each stage of the disease. This enables the elucidation of pathogenic mechanisms, the search for biomarkers for early detection, and the determination of the therapeutic effects of new drugs. However, animal models of central nervous system diseases require euthanasia for brain sampling, so changes over time can be followed with only a mass-producible model, such as rodents, and it is difficult to follow the same individuals in such a research system. In addition, there is a problem with species differences between preclinical studies in rodents and clinical studies in humans, and good performance in rodents is often not conducive to clinical studies.

We proposed a brain evaluation method using an MRI system, which can evaluate the structure and form of the brain and the function of the brain using a technique called functional MRI (fMRI). Functional MRI can estimate the local activation of the brain and areas that cooperate with each other on large-scale networks based on changes in the contrast of MR images obtained over time. Functional MRI can also be used to non-invasively and repeatedly measure brain function in diseased animals, thus solving the problems described above. Furthermore, the greatest advantage of MRI-based brain function research is that if fMRI makes it possible to detect abnormalities in brain function in disease primate models, the results can be directly introduced into clinical practice, where tissue sampling is often difficult for central nervous system diseases in medical situations. Therefore, MRI is a suitable tool for exploring the findings obtained using an invasive method in experimental animals that can be detected in a clinical setting, making it a good match for preclinical studies using marmosets.

Therefore, brain function studies using fMRI in genetically modified marmosets are extremely important for extrapolating clinical research data. However, only few fMRI studies of marmosets have been reported, especially concerning large-scale brain networks^7–9^, which are said to be altered in humans with higher brain dysfunction. Large-scale networks are networks constructed among several co-working brain regions that are measured using special fMRI known as resting-state fMRI (rsfMRI), and are performed in a resting state (not doing anything in particular); a large-scale network is also called a resting-state network (RSN). In humans, many institutions and researchers have investigated RSNs using various methods, and currently seventeen networks have been identified^10,11^. These networks are reportedly altered in neurodegenerative diseases^12–15^, suggesting that RSNs may serve as biomarkers in diseased model marmosets. Similarly, by conducting multifaceted analyses in many facilities, subjects, and researchers, more reliable RSNs can be identified in marmosets, and will contribute to promoting future brain function research.

This study aimed to identify RSNs stably observed in awake and normal marmosets by evaluating commonality and their inter-individual variance, and to detect brain regions involved in the composition of the RSNs to understand the species differences that occur between humans and marmosets. In this study, we conducted an analysis using 100,000 time-series data points, the data volume of which was larger than that in previous reports, obtained by trimming eight high-quality data points to ensure the detection of RSNs.

## Results

### Evaluation of rsfMRI data quality

**Figure 1:**
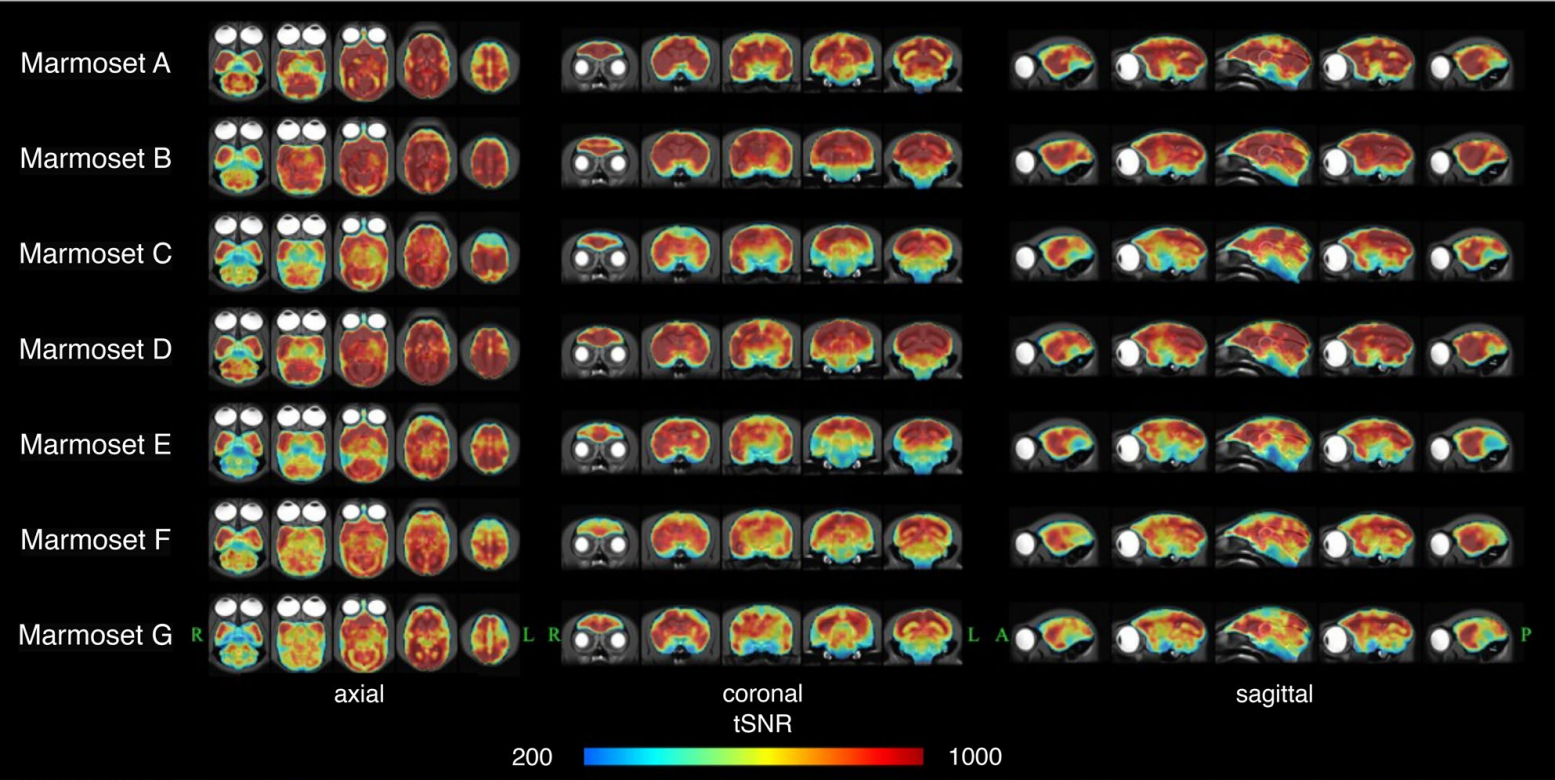
The temporal signal-to-noise ratios (tSNR) in each subject. This figure shows the mean tSNR for each subject. For each subject, a high-resolution T2 weighted imaging image overlaid with a tSNR image is shown in three sections: axial, coronal, and sagittal.

Appropriate data for group analysis, in which individual anatomical variation was removed and noise caused by body movement were obtained with image distortion correction and head motion correction, resulting tSNR showed extremely high values (Fig. 1). The tSNR maps were measured in a single session and averaged over 12 sessions for each participant. Almost all voxels in the whole brain of all participants had a tSNR > 200, and a large number of voxels had a tSNR > 500.

The results of the image distortion correction and head motion correction are shown in Supplemental Figs. 1, 2 and 3. Supplemental Figs. 1 and 2 shows images before and after distortion reduction and after registration to the template image in all individuals. It can be seen that distortion and registration corrected for morphological individual differences and increased the absolute BOLD value. Supplemental Fig. 3 shows the six movement parameters (translations and rotations) calculated for each subject and the ex vivo brain. Head motion in the rsfMRI scans of each subject was estimated using realignment. For translational motion, the tendency for motion in the z-direction to be larger than that in the other two directions was common to all subjects (Supplemental Fig. 3A). The maximum values of the translations were relatively stable at 0.08674 mm, 0.1258 mm, and 0.1941 mm in the x-, y-, and z-directions, respectively. For rotational motion, pitch tended to be slightly larger than roll and yaw (Supplemental Fig. 3B). The maximum values of the pitch, roll, and yaw rotational motions in the rsfMRI data of all subjects were 0.015210°, 0.006158°, and 0.006399°, respectively. All these values were very small, but denoising with CONN eliminated the noise from the blood-oxygen-level-dependent signal.

### Resting-state networks detected in most of the common marmoset

Sixteen networks were detected in more than four animals as SIGNALs and UNKNOWNs. These RSNs were named based on previous studies, and RSNs that have not been reported previously were named based on human studies and the regions constructing these RSNs. They were roughly divided into three groups based on information in the spatial map: cortical, visual, and subcortical network groups. The cortical network group contained eight networks, which were labeled as follows: RSN1, default mode network; RSN2, dorsomedial somatomotor network; RSN3, ventral somatomotor network; RSN4, dorsal frontal network; RSN5, central frontal network; RSN6, auditory network; RSN7, temporal pole network; and RSN8, temporal lobe network. The visual network group contained five networks that were labeled as follows: RSN9, primary visual network; RSN10, dorsal caudal visual network; RSN11, right ventral visual network; RSN12, left ventral visual network; and RSN13, lateral visual network. The subcortical network group contained three networks, labeled as follows: RSN14, limbic network; RSN15, basal ganglia network; and RSN16, cerebellar network.

**Figure 2:**
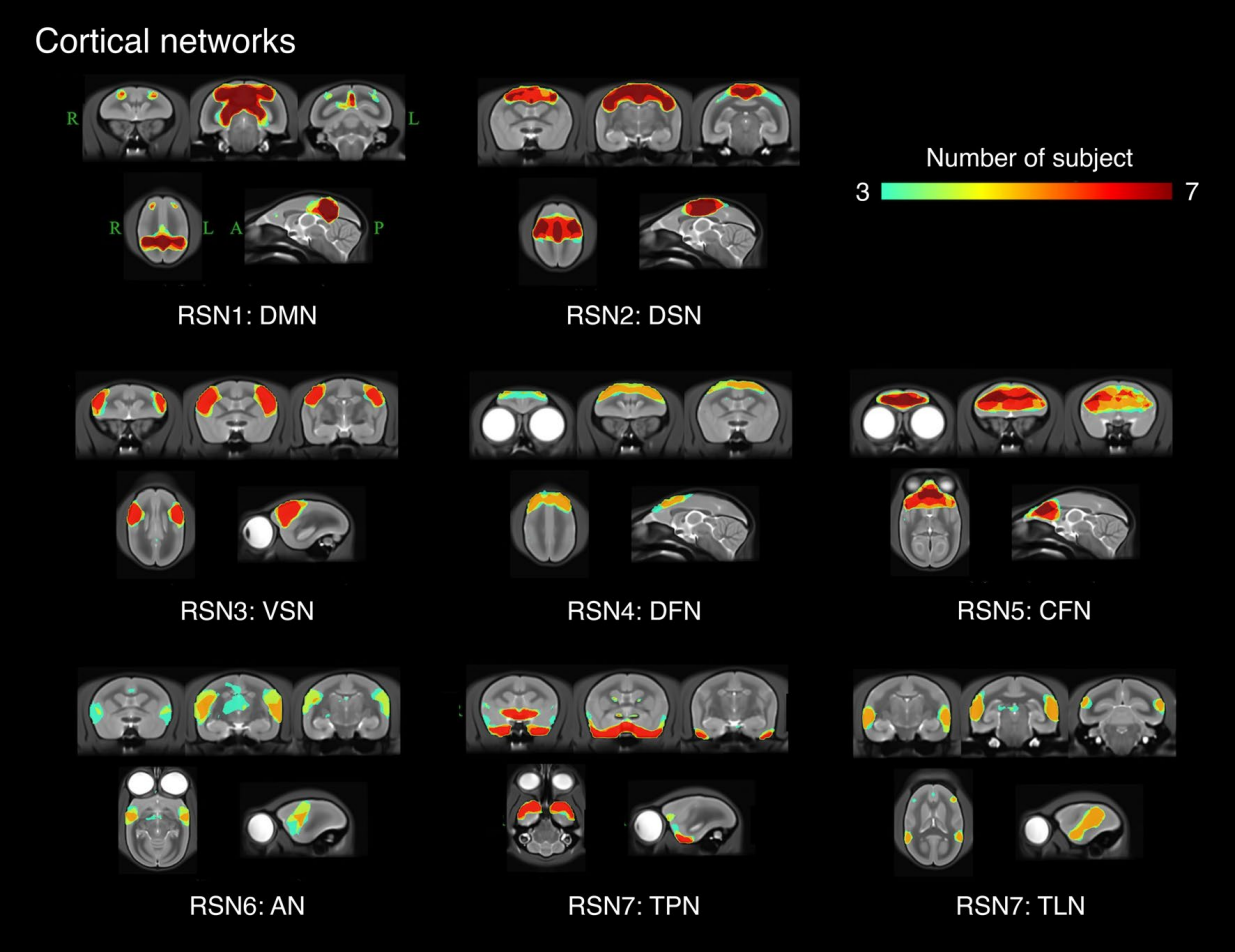
Merged cortical network image of subject-ICA from 7 marmosets. The number of individuals for which a network was detected in each region is shown with varying color tones. RSN1: DMN, default mode network; RSN2: DSM, dorsomedial somatomotor network; RSN3: VSN, ventral somatomotor network; RSN4: DFN, dorsal frontal network; RSN5: CFN, central frontal network; RSN6: AN, auditory network; RSN7: TPN, temporal pole network; RSN8: TLN, temporal lobe network.

**Figure 3:**
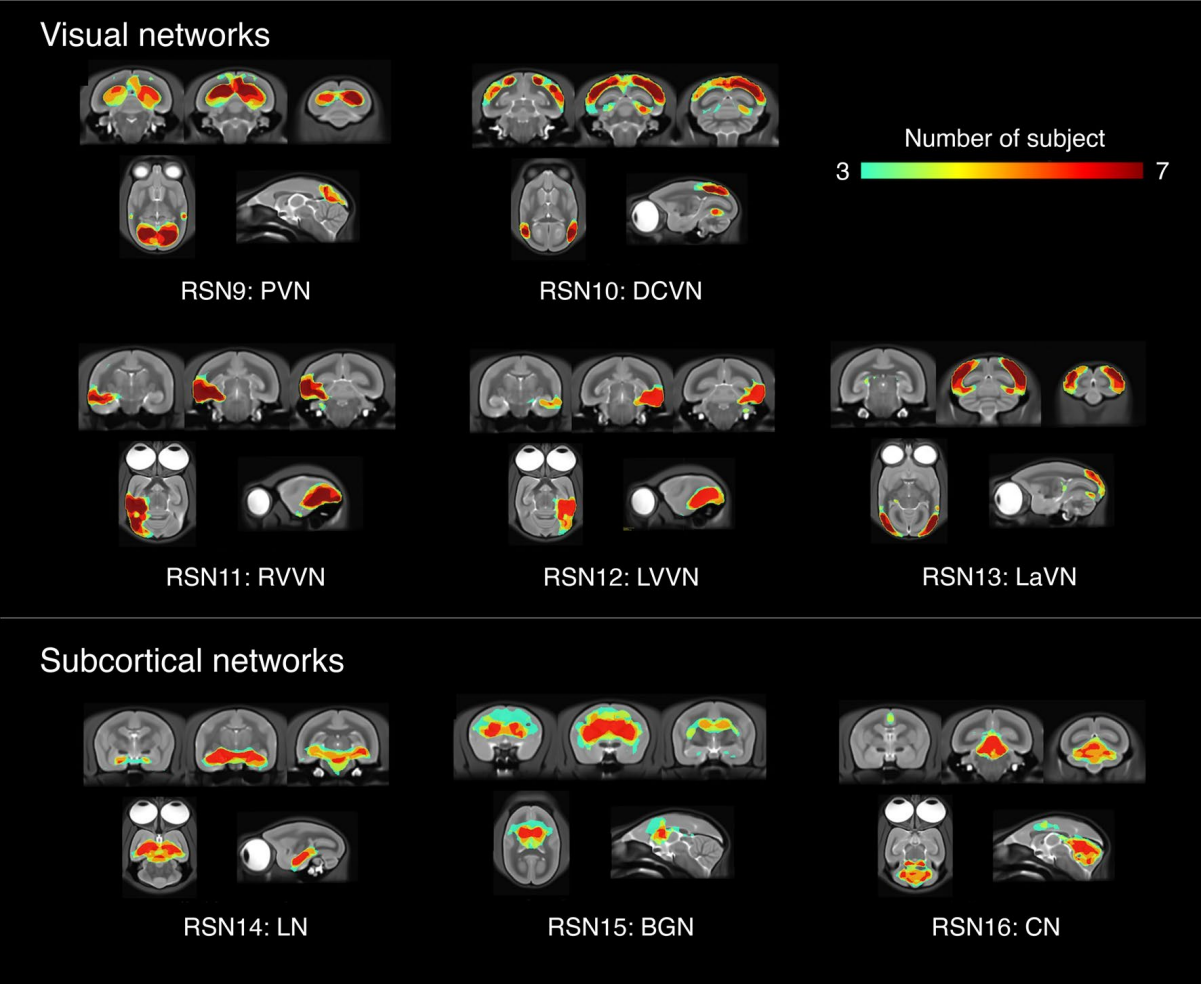
Merged visual and subcortical network image of subject-ICA from 7 marmosets. The number of individuals for which a network was detected in each region is shown with varying color tones. RSN9: PVN, primary visual network; RSN10: DCVN, dorsal caudal visual network; RSN11: RVVN, right ventral visual network; RSN12: LVVN, left ventral visual network; RSN13: LVN, lateral visual network. RSN14: LM, limbic network; RSN15: BGN, basal ganglia network; RSN16: CN, cerebellar network.

Figures 2 and 3 show the merged images of the independent component analysis (ICA) of each subject. Dorsal frontal, auditory, and temporal lobe networks were not observed in several marmosets; however, the other networks were observed in almost all marmosets.

### Analysis of brain regions comprising RSNs detected using ICA

**Figure 4:**
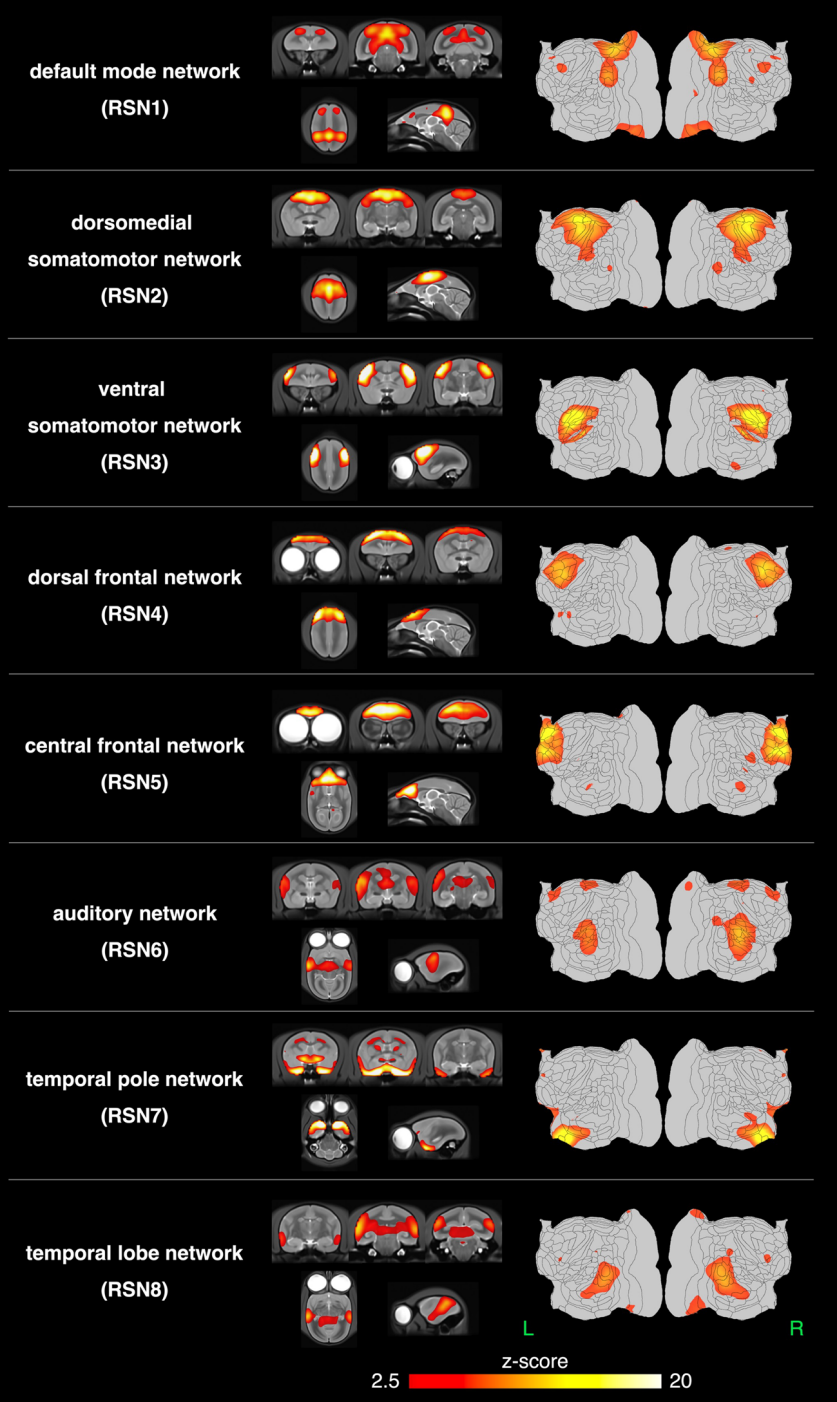
The spatial maps of the 8 resting-state networks classified as the cortical network group. The cortical network group contained eight networks, labeled as follows: RSN1, default mode network; RSN2, dorsomedial somatomotor network; RSN3, ventral somatomotor network; RSN4, dorsal frontal network; RSN5, central frontal network; RSN6, auditory network; RSN7, temporal pole network; and RSN8, temporal lobe network. The z-score threshold (z = 2.5) was used to visualize the z-score maps. The z-score maps were overlaid on a template T2WI and flat map.

Figure 4 shows the spatial maps of the RSNs classified as the cortical network group in the template T2WI and flat map. See Supplemental Fig. 4 and Supplemental Tables 1 and 2 for the flat map used in this study, the region name, and abbreviation. The major regions detected in each component using group-ICA are as follows:

- RSN1: default mode network
  A6DR, A19M, LIP, MIP, OPt, PG, PGM, V3A, VIP, SC, IC, caudal parts of the Thal, and HipF.
- RSN2: dorsomedial somatomotor network
  A1/2, A23a-b, A24b-d, A3a, A3b, A31, A4ab, and PE.
- RSN3: ventral somatomotor network
  A1/2, A3a, A3b, GI, S2PR, S2PV, S2I, and S2E.
- RSN4: dorsal frontal network
  A6DC, A6DR, A6M, A8b A8aD, A8aV, and A8C
- RSN5: central frontal network
  A9, A10, A11, A24a-b, A32, A32V, and A45
- RSN6: auditory network
  AuA1, AuAL, AuCL, AuCM, AuCPB, AuML, AuR, AuRM, AuRPB, AuRT, AuRTL, and AuRTM.
- RSN7: temporal pole network
  A35, A36, Apri, AuRPB, Ent, OPAl, OPro, STR, TE1, TE2, and TF.
- RSN8: temporal lobe network
  V4T, V5, MST, FST, PGa-IPa, TE2, and TE3.

**Figure 5:**
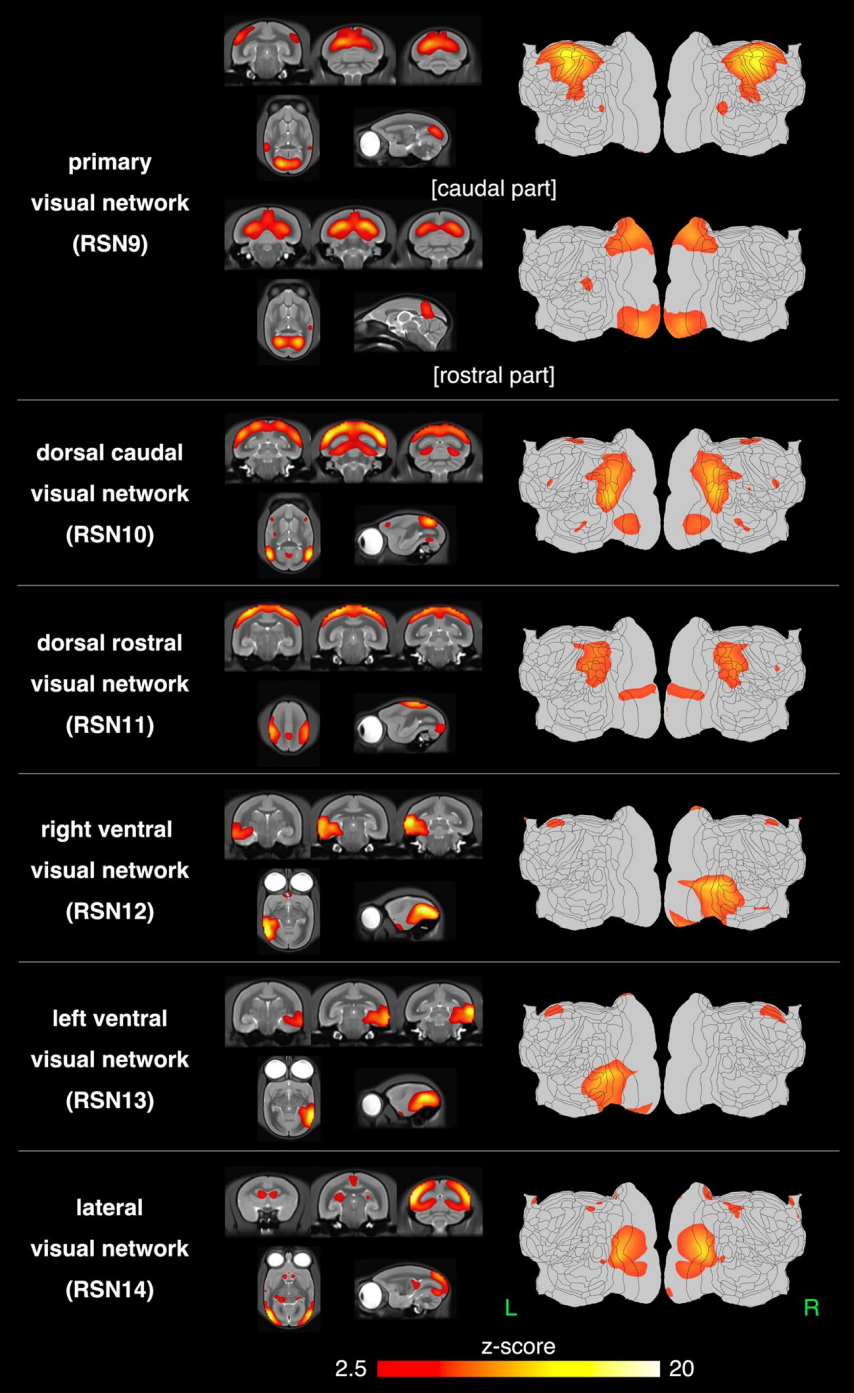
The spatial maps of the 5 resting-state networks classified as the visual network group. The visual network group contains six networks labeled as follows: RSN9, primary visual network; RSN10, dorsal caudal visual network; RSN11, right ventral visual network; RSN12, left ventral visual network; and RSN13, lateral visual network. The primary visual network (RSN9) consists of two separate components. The z-score threshold (z = 2.5) was used to visualize the z-score maps. The z-score maps were overlaid on a template T2WI and flat map.

Figure 5 shows the spatial maps of the RSNs classified as the visual network group in the template T2WI and flat map.

- RSN9: primary visual network
  In this study, these two components peaked at V1. These components were defined as the caudal and rostral parts, according to the position of the peak in V1. The caudal part included V1, V2, A24a–d, parts of FST, MST, OPt, PG, TPt, V4T, and V5. The rostral part included V1, V2, A23V, A19M, ProSt, A29a–c, parts of A30, PGM, and V3.
- RSN10: dorsal caudal visual network
  The peak of this network was located on the caudal side, and in the parts of V2 and V3. AIP, MIP, LIP, OPt, PE, PEC, PFG, PG, V2, V3, V4T, V5, V6, V6A, and VIP.
- RSN11: right ventral visual network
  V2, V3, V4, V4T, V5, FST, TE3, TEO, TFO, and TLO.
- RSN12: left ventral visual network
  This RSN was located in the ventral cortex regions of the left brain hemisphere and indicated a spatial distribution that mirrored that of the right ventral visual network.
- RSN13: lateral visual network
  The RSN can be divided into the cortical and subcortical regions. In the cortical regions, RSN was composed primarily of A19DI, V1, V2, V3, and parts of A25, A31, V4, and V4T. However, the lateral part of the visual cortex was included in the RSN. In subcortical regions, this RSN covered part of the SC, Thal, and LGN. The subcortical regions in the frontal region include parts of the Sep and Cd.

**Figure 6:**
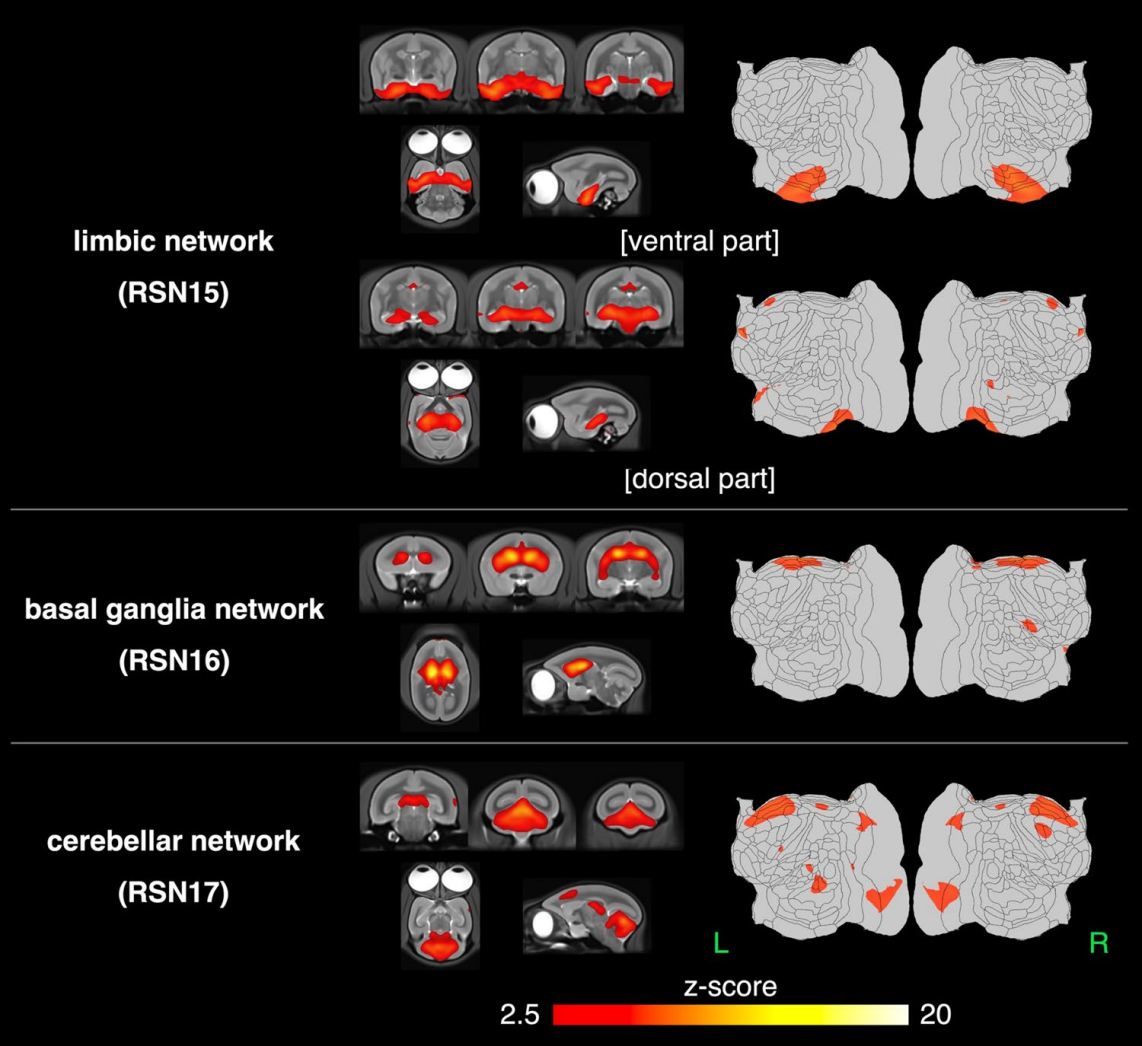
The spatial maps of the 3 resting-state networks classified as the cortical network group. The subcortical network group contains three networks, labeled as follows: RSN14, limbic network; RSN15, basal ganglia network; and RSN16, cerebellar network. The limbic network (RSN15) was detected in two separate components. A z-score threshold (z = 2.5) was used to visualize the z-score maps. The z-score maps were overlaid on a template T2WI and flat map.

Figure 6 shows the spatial maps of the RSNs classified as the subcortical network group in the template T2WI and flat map. In these RSNs were mainly detected in subcortical regions.

- RSN14: limbic network
  The dorsal part; HipF, SNR, and red nucleus, the oculomotor nucleus, intermediate nucleus of the lateral lemniscus, medial geniculate nucleus, reticulotegmental nucleus of the pons, principal sensory trigeminal nucleus, and part of the Thal.
  The ventral part; A35, A36, Ent, TE2, TE3, TEO, TF, TFO, TL, and TLO.
- RSN15: basal ganglia network
  Cd, Pu, Acb, Cl, GP, Sep, and Thal.
- RSN16: cerebellar network
  Cer.

## Discussion

In this study, we used more than 100,000 time-course image volumes to detect marmoset RSNs and evaluate the commonality and inter-individual variance of these RSNs. Careful preprocessing was performed, and the quality was assessed by measuring the tSNR. The tSNR measured in this study was > 200 in almost all voxels in the entire brain, and a large number of voxels had a tSNR greater than 500 in all subjects. According to a previous study, to detect activation with an effect size of 0.5% to a threshold of P = 5 × 10^−10^ with 150 volumes of rsfMRI data (= 1 session), a tSNR of approximately 200 is warranted according to the theory^16^. In addition, to detect activation with an effect size of 0.2% to a threshold of P = 5 × 10^−10^ with 150 volumes of rsfMRI data, a tSNR of approximately 500 was required. Therefore, the rsfMRI data used for the ICA in this study can be considered to have a sufficiently high tSNR to detect activation. Because of single-ICA for denoising, 8781 components were detected, with an average of 12.2 components in a single-ICA. In addition, we performed ICA using all rsfMRI data following denoising to investigate the RSNs.

### Inter-individual evaluation of RSNs

In the detection and evaluation of RSNs and their inter-individual variation, 16 RSNs were detected and 13 RSNs were observed in almost all marmosets. Table 1 compares the networks observed in previous studies on marmosets, humans, and macaques. Ten of these RSNs (default mode network, dorsomedial somatomotor network, ventral somatomotor network, central frontal network, primary visual network, dorsal caudal visual network, right ventral visual network, left ventral visual network, basal ganglia network, and cerebellum network) were similar to RSNs reported in the previous studies in marmosets and also observed in almost all individuals in this study^7,8,17^. It has been suggested that these networks are RSNs commonly observed in resting-state common marmosets. Although dorsal frontal, auditory, and temporal lobe networks have been reported as similar networks to those commonly observed in previous studies^17^, they were observed in comparatively fewer participants in this study. These networks may depend on the participants or their mental conditions. However the remaining three networks (lateral visual network, temporal pole network, and limbic network) were not reported in the previous marmoset study, they were detected with careful preprocessing, ICA denoising, and ICA signal selection in this study. Their signal peak was recorded in the cerebral cortex area, not in the cerebral fluid or major vessel area. These features are consistent with the RSN features detected in ICA, as reported in previous studies^18^, therefore, these RSNs also appeared to be the RSNs of common marmosets. Furthermore, 11 networks (default mode network, dorsomedial somatomotor network, ventral somatomotor network, auditory network, temporal pole network, temporal lobe network, primary visual network, lateral visual network, limbic network, basal ganglia network, cerebellum network) were reported as RSNs in previous studies in humans and/or macaques^19–29^. All 11 networks including three RSNs which have not been reported in marmosets (lateral visual, temporal pole, and limbic networks), were detected in this study. This suggests that these three networks are common in marmosets. In contrast, the dorsal frontal network, central frontal network, dorsal caudal visual network, right ventral visual network, and left ventral visual network have not been reported in humans and macaques but have only been reported in previous marmoset studies. In human studies, the saliency network, attention network, and executive network are known as cortical RSNs. The dorsal frontal network and central frontal network detected in this study may be equivalent to these networks. Further rsfMRI studies with appropriate stimulation or tasks are required to investigate which network is equivalent to the saliency network, attention network, or executive network.

**Table 1:** A comparison of networks observed in previous marmoset, human, and macaques studies.

RSN	RSN1 DMN	RSN2 DSM	RSN3 VSN	RSN4 DFN	RSN5 CFN	RSN6 AN	RSN7 TPN	RSN8 TLN
This study	✔	✔	✔	△	✔	△	✔	△
Previous study	✔	✔	✔	✔	✔	✔		✔
Human/Macaque	✔	✔	✔			✔	✔	✔

RSN	RSN9 PVN	RSN10 DCVM	RSN11 RVVN	RSN12 LVVN	RSN13 LVN	RSN14 LN	RSN15 BGN	RSN16 CN
This study	✔	✔	✔	✔	✔	✔	✔	✔
Previous study	✔	✔	✔	✔			✔	✔
Human/Macaque	✔				✔	✔	✔	✔

Networks in humans are from Yeo et al.^11^ and Beckmann et al.^29^. Macaques are from Hutchison et al.^21^ and Yacoub et al.^26^. Marmosets are from Belcher^7^, Hori et al.^8,17^.

In this study, cortical visual networks were detected, and the dorsal caudal visual network, right ventral visual network, and left ventral visual network were reported only in marmosets. These networks were reported as “higher-visual network”^8^. Since the percentage of the visual cortex in all cortical regions in the common marmoset is higher than that in other primates^30–35^, these networks might be specific to marmosets. The physiological functions of these networks and others need to be assessed through further rsfMRI experiments with appropriate stimuli and conditions, and how the networks are altered. Filtered cells indicate the networks detected in this study and previous human or macaque studies but not detected in previous marmoset studies. The check symbol indicates the detected RSNs in this study or a previous study, and the triangle symbol indicates RSNs detected in major, but not all, marmosets in this study. RSN1, default mode network; RSN2, dorsomedial somatomotor network; RSN3, ventral somatomotor network; RSN4, dorsal frontal network; RSN5, central frontal network; RSN6, auditory network; RSN7, temporal pole network; RSN8, temporal lobe network; RSN9, primary visual network; RSN10, dorsal caudal visual network; RSN11, right ventral visual network; RSN12, left ventral visual network; RSN13, lateral visual network; RSN14, limbic network; RSN15, basal ganglia network; RSN16, cerebellum network.

### Comparison of RSNs with previous marmoset, macaque, and human studies

#### Default mode network (DMN)

The prefrontal regions detected in this study were slightly different from the DMN detected in previous studies; however, almost all regions were similar to those detected in previous marmoset studies. Most previous studies have reported A8, such as A8C and A8aD, as a constituent region of the DMN in addition to A6DR^7,9,36^. The PFC of the DMN that we detected had A6DR as the major region, and parts of A6DC, A8aD, and A8b were also present. This difference may have been caused by differences in the template and atlas employed.

Furthermore, the DMN in this study included not only cortical regions, but also subcortical regions. Our data showed that the SC, IC, and caudal parts of the Thal and HipF were also included in the RSN7. The hippocampus was mentioned in previous studies as a subcortical region closely related to the DMN. The functional relationship between the DMN and hippocampus has also been reported in human, macaque, and rat models^37–39^. On the other hand, there have been conflicting reports regarding the DMN and SC in marmosets, with Hori et al. arguing that it is one of the constituent regions of the DMN^17^, and Ghahremani et al. arguing that the DMN in marmosets that have been reported to contain SC is actually a different network^40^. In this study, we classified DMN with reference to previous studies, but as mentioned in the Introduction, it is necessary to conclude from multifaceted studies. The functional relationship between the IC and DMN also requires additional investigation.

#### Temporal pole network

RSN7 exhibits a peak in the temporal pole area. Recently, the temporal pole network has been reported as an RSN in humans and macaques^26,28^. The temporal pole corresponds to the most anterior part of the anterior temporal lobe^23^. Although the temporal pole network has been poorly reported in animal species, it might be a common RSN in primates, including common marmosets. The reason for the few reported cases in this network could be the location of the temporal pole close to the paranasal sinuses, which is a region prone to distortion and loss of signal intensity due to EPI.

The anterior temporal lobe has functions related to semantic memory^23^, and neurodegeneration reportedly occurs in the temporal pole and affects the functional connections associated with the temporal pole in semantic variant primary progressive aphasia (svPPA), which is classified as a neurodegenerative disease^41,42^. Thus, this network could be useful in the evaluation of semantic memory and in investigating the functional effects of diseases such as svPPA.

#### Lateral visual network

A previous study on RSNs in common marmosets reported several similar visual networks, named higher order visual networks^17^, however, the primary visual cortex was not included in these higher order visual networks. On the other hand, the lateral visual network in this study was mainly constructed with the primary visual cortex. Furthermore, this network included some subcortical cortices, such as the SC and LGN, in addition to the lateral parts of the primary visual cortex. Therefore, we judged these to be different networks.

Examination of the relationship between the visual cortex and these subcortical regions revealed that the visual pathway is a candidate. In common marmoset brains, previous studies on the visual system using tracer injection showed that visual cortices are associated with the LGN and pulvinar^43^, which indicates that the visual cortices and subcortical cortices are coupled. Based on previous neuroscience studies, this network may be significantly involved in the visual pathways of the common marmoset.

#### Limbic network

The limbic network includes the hippocampal formation, Amy, and parts of the brainstem. The hippocampus and amygdala are parts of the limbic system^44^, and a functional relationship between the limbic system and brainstem in the human brain has been reported^45^. The limbic network which consists of the limbic system and brainstem has been reported to be a human RSN^22^. Therefore, the network used in this study may be similar to the human limbic network. A previous study showed that this network is associated with cognition and other functions^45^. In addition, abnormalities in this network can result in schizophrenia and other functional disorders^46–48^. Therefore, the limbic network may be highly useful as an indicator for examining brain functions such as cognition, and as a biomarker for evaluating diseases such as schizophrenia.

## Limitation

This study had several limitations. First, although we performed rigorous training on the subjects before the MRI experiment, we observed that the subjects occasionally closed their eyes during scanning. According to a previous study, the definition of “rest” did not require the opening and closure of the subject's eyes^49^. Therefore, the MR images used in this study were obtained from rsfMRI data. This is a common issue with awake rsfMRI^7^. Second, the number of ICA dimensions is a limiting factor in the image analysis. A trade-off exists between the number of ICA dimensions and the observed network^26^. If the dimensionality of ICA was changed, the results will vary. Finally, it should be noted that this study was limited by the sex and age of the participants. A previous study reported that the volume of gray and white matter in common marmosets increased or decreased with age^50^. It has also been reported that brain volume and structural connectivity vary with age^51^. In the future, we plan to investigate RSNs in common marmosets across a wide age range, regardless of sex.

## Conclusion

Many of the RSNs in this study had a spatial distribution similar to that of RSNs in humans and/or macaques. Thus, the common marmoset might be similar in brain structure and resting-state function to these primates. This observation is important for understanding brain evolution. In addition, we present new findings regarding RSNs in common marmoset brains. We examined the temporal pole, lateral visual network, and limbic networks, which have not been previously reported in common marmosets. Furthermore, these RSNs have been reported to be associated with neurodegenerative diseases such as AD and PD. In the Brain/MINDS project, common marmoset models of neurodegenerative diseases have been developed using genetic modification techniques. Therefore, our data are useful for evaluating the functional effects of these diseases using common marmosets.

## Materials and methods

### Animal care and use committees

This study was approved by the Animal Experiment Committee at the RIKEN Center for Brain Science (CBS) and was conducted in accordance with the Guidelines for Conducting Animal Experiments of the RIKEN CBS, and all experiments were performed in accordance with the ARRIVE guidelines.

#### Animals

We included seven healthy common marmosets (4 males and 3 females; 4.29 ± 1.31 years old; 380–450 g) in this study. Before the main experiments, simple brain structural magnetic resonance images were obtained for all animals to ensure that none of them had brain structural anomalies. All the animals underwent surgery to attach a head post to the cranial bones. Details of the surgery are described in a previous study^52^. The headpost was fixed using a custom-made instrument to prevent head movements. The details of the immobilization are described in a previous study^52^.

#### Acclimatization to rsfMRI

After the headpost was attached, acclimatization to the rsfMRI was performed. Acclimatization was divided into two stages: (A) acclimatization to the experimental environment and (B) acclimatization to the MRI system. In Stage A, the marmosets were gradually acclimatized to the experimental environment in four steps. Acclimatization days were set aside for 2–3 days per week, with one step each week.

A-1: Acclimatization to the experimental environment: The marmosets were placed in the carry cage, which was in the MRI room without fixing their bodies and heads for 30 min before step A-2–A-4.

A-2: Acclimatization to fixed equipment: The marmosets were placed on the scan bed and fixed in a prone position using body-fixing tools. 30, 60, and 90 min per day for a total of 3 days in the first week.

A-3: Acclimatization to immobilization with the head post Marmosets were immobilized with a custom-made headpost fixing instrument. They were also fixed with the body-fixing tools used in Stage A-2. 30, 60, and 90 min per day for a total of three days in the second week.

A-4: Acclimatization to actual fixing during MRI scanning: In addition to stage A-3, earplugs and a receiver coil were placed for 90 min in three days in the third week.

All individuals were stable in the experimental environment and progressed to Stage B. In Stage B, the marmosets were gradually acclimatized to the MRI system in two steps.

B-1: Acclimatization to a static magnetic field: The marmosets were placed inside the MRI bore for the same duration as the actual scanning time and acclimatized to a static magnetic field with earplugs and a receiver coil. 30, 60, and 90 min per day for a total of three days in the forth week.

B-2: Acclimatization to the scanning environment: The marmosets were fixed on a bed and placed inside the MRI bore under the same condition in Stare B-2, where they were kept in a resting state while listening to the sounds generated during rsfMRI. 30, 60, and 90 min per day for a total of three days in the fifth week.

During Stage B, the marmosets fasted for at least 4 h before training to avoid aspiration following nausea and vomiting caused by the magnetic field. Marmosets were constantly monitored using an infrared camera. The maximum duration of each step was set to 90 min, considering the physical and mental burden on the marmosets. This was the approximate time required to acquire all the MR image data necessary for the analysis when the rsfMRI data were acquired for 60 min. Marmosets were rewarded with highly palatable food at the end of each acclimatization session to reduce their mental burden. After acclimatization, all the marmosets scored 1 or 2 on the behavioral assessment scale^53^ in the rsfMRI scan environment.

#### Data acquisition

All scans were performed using a 9.4 T MRI Scanner (Bruker, Biospec94/30) and ParaVision 6.0.1 (Bruker, Billerica, USA) with a gradient set capable of 660 mT/m. For transmission, a quadrature detection coil (inner diameter = 154 mm) was used as the volume coil and an 8-channel phase-array receiver coil for the common marmoset (Takashima Seisakusho Co., Ltd., Japan) was used. Resting state fMRI scanning was carried out using gradient-echo echo planar imaging (EPI) sequence (repetition time [TR] = 2000 ms, echo time [TE] = 16 ms, flip angle = 65°, field of view [FOV] = 42.0 × 28.0 mm, matrix size = 60 × 40, 52 coronal slices, resolution = 0.7 mm isotropic, number of excitations [NEX] = 1, 155 time-course image volumes, duration = 5 min 10 s). Twelve sessions, which consisted of approximately 60 min of scanning data and 1,800 time-course image volumes, were obtained in one experiment, and the total rsfMRI data for all subjects were 108,000 time-course image volumes (60 h of data scanning, number of experiments was 6–10 for each individual). In addition, T2 weighed imaging (WI) was scanned as a structural image (TR = 4331 ms, TE = 15 ms, flip angle = 90°, FOV = 42.0 × 28.0 mm, matrix size = 120 × 80, 52 coronal slices, resolution = 0.35 × 0.35 × 0.7 mm, NEX = 1). To correct the distortions caused by the EPI sequence, two sets of spin-echo EPI sequences with opposite phase-encoding directions (head–foot and foot–head) were scanned (TR = 5000 ms, TE = 22 ms, flip angle = 90°, FOV = 42.0 × 28.0 mm, matrix size = 60 × 40, 52 coronal slices, resolution = 0.7 mm isotropic, NEX = 1, 6 time points). Furthermore, ex vivo brains were scanned for 72 sessions of rsfMRI, T2WI, and two sets of spin-echo EPI data to evaluate head motion.

During the MRI scan, a pulse oximeter was attached to the marmoset’s calf, and oxygen saturation and heart rate were constantly monitored. These values were recorded every 10 min. To prevent a drop in body temperature, a tube with warm water running through it was wrapped in cloth and placed on the subject’s abdomen during the MRI scan. In addition, an infrared camera was placed in front of the subject’s face for constant observation during the MRI scan. If abnormal vital signs were recorded continuously for 10 min, the experiment was terminated to consider the physical condition of the marmoset. Any contrast agent to enhance the MR signal was not used in this study.

#### Image preprocessing and RNS detection

**Figure 7:**
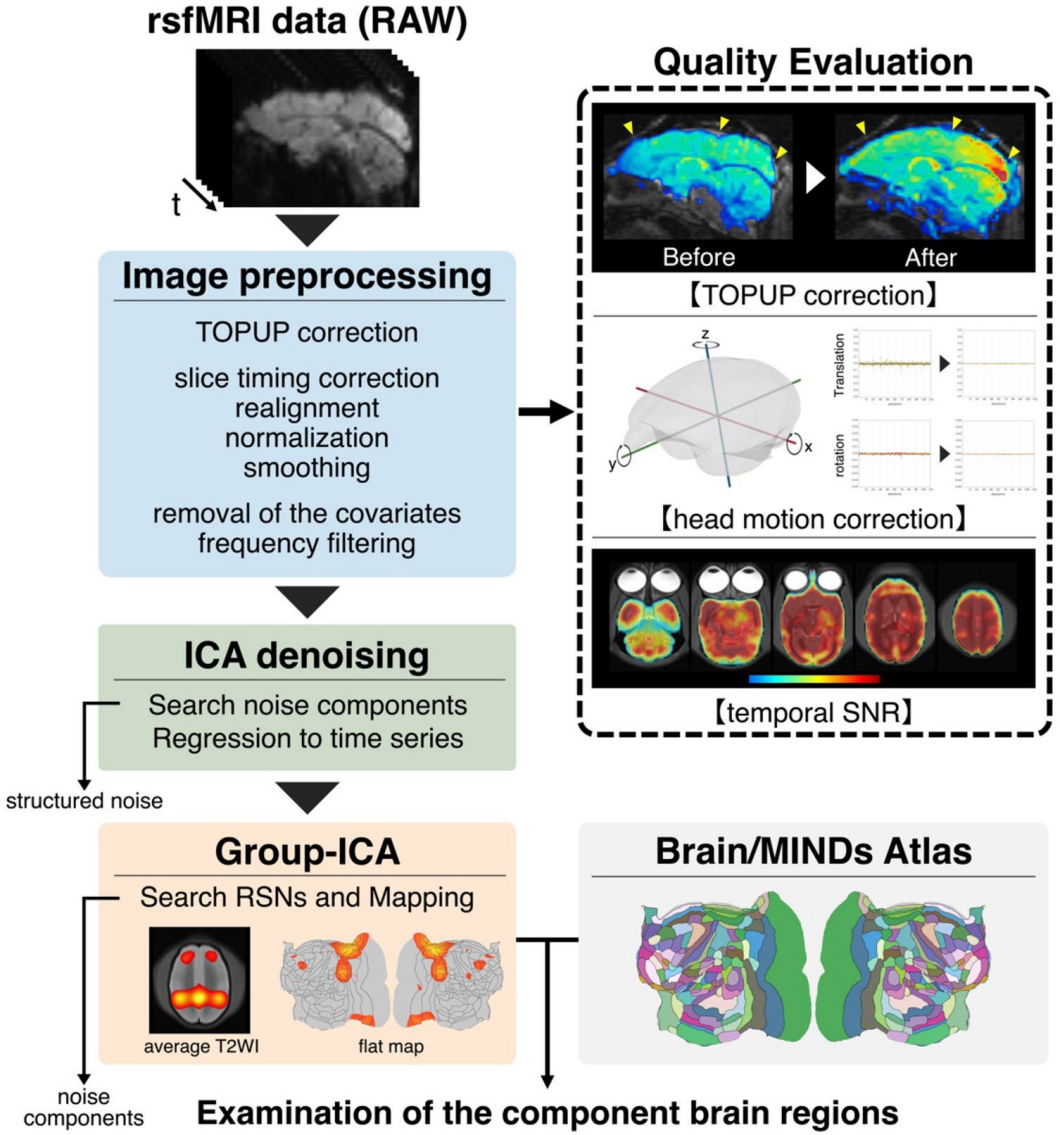
Flowchart of the image preprocessing, quality evaluation, and resting-state network (RSN) analysis. This figure shows the flowchart from image preprocessing to the examination of RSNs in this study. First, image preprocessing was performed and all the resting-state functional magnetic resonance imaging (rsfMRI) data were normalized to the template data. To evaluate the quality of the fMRI data, correction of image distortion and head movement were confirmed. In addition, temporal signal-to-noise ratio (tSNR) per session was calculated and evaluated. Second, independent component analysis (ICA) for each session (single-ICAs) was performed and the noise component was removed. Third, ICA (group-ICA) with all data was performed, and SIGNAL components were RSNs. Finally, RSNs were mapped to T2 weighted imaging and flat maps, and the brain regions comprising the network were examined.

Figure 7 shows a flowchart of the image preprocessing, quality evaluation, and RSNs analysis. Before analyzing the RSNs in the common marmoset, the raw MRI data were preprocessed. The TOPUP tool in the FMRIB Software Library (FSL) was used to correct distortions in the head–foot and foot–head directions^20,54–56^. To ensure a more accurate and effective correction, the voxel size was pseudo-changed from 0.7 mm isotropic to 3.5 mm isotropic, which is equivalent to humans using the Statistical Parametric Mapping 12 tool (SPM12)^56^. After confirming that the image distortion was successfully rectified, realignment, slice timing correction, normalization, and smoothing were performed using SPM12. Smoothing was performed using FWHM = 2 voxels. The magnitude of FWHM during smoothing was determined based on previous studies^57^. Subsequently, denoising and frequency filtering were performed using CONN^58^. Denoising refers to the removal of covariates, such as white matter and cerebrospinal fluid signals. In addition, head motion was eliminated during this step^59^. A frequency band > 0.001 Hz was used for frequency filtering. To evaluate the quality of the rsfMRI data, the temporal signal-to-noise ratio (tSNR) was calculated for each session. To calculate and evaluate tSNR, previous studies were used to examine the relationship between tSNR and fMRI scan duration^16^. The tSNR was calculated using MATLAB (Version 9.10, MathWorks Inc.) with using formulas presented in a previous study^16^. To examine the brain regions comprising the RSNs, MATLAB was used to match the spatial distribution of the RSNs (z-score threshold at z = 2.5) with the BMA atlas.

Independent component analysis (ICA) was performed using the Multivariate Exploratory Linear Optimized Decomposition into Independent Components (MELODIC; version 3.15) module in FSL^55^ to detect RSNs in marmosets. First, demean and variance normalization were carried out on the time series of each voxel^60^, and single-ICA was performed for each session. The number of dimensions was set for automatic estimation. Noise components were selected and regressed from the preprocessed MRI data using the FSL tool (fsl_regfilt) and regressed on the time course of each preprocessed rsfMRI dataset based on previous studies^18^.

Subsequently, subject-ICA and group-ICA were performed to evaluate the inter-individual variance of RSNs and understand the brain regions involved in the composition of these RSNs, respectively. The optimal dimensions of the ICA were decided through the 7 times implemented ICA analysis with different dimension numbers (10, 15, 20, 25, 30, 35, and 40). The 25 dimensions were selected because these dimensions could detect the most bilaterally symmetrical network in our data.

In subject-ICA, each component was classified as SIGNAL, UNKNWON, or NOISE. To avoid subjective selection and to objectively classify the components, a previously reported classification method was employed^18^. In this method, the time series data of each component in each session was obtained using the FSL tool (dual regression), and the power spectrum of the time series data was calculated using MATLAB because the special map obtained the ICA analysis, time series data, and power spectrum data. In the components classified as SIGNAL and UNKNWON, the RSNs of each individual were compared and the same networks and networks detected by more than four individuals were defined as commonly observed RSNs.

In the ICA group, cortical and subcortical region atlas data from the Brain/MINDS Marmoset Reference Atlas (BMA; http://brainatlas.brain.riken.jp/marmoset/modules/xoonips/listitem.php?indexid=66)^61–63^ were used to examine the constituent regions of the RSNs. This atlas was registered to the template data using Advanced Normalization Tools (ANTs)^64^ to organize their spatial locations using rsfMRI data. After registration, there were 214 regions, including both cortical and subcortical regions. To visualize the results of group-ICA, the average template T2WI data and flat map were merged with the group-ICA data. Average template T2WI was generated using in vivo MRI data from 216 common marmosets. The average template T2WI was spatially aligned with the template data using FSL FMRIB’s Linear Image Registration Tool^65,66^, and overlaid with the component data. The component data and BMA atlas were also registered to a flat map using ANTs^64^ and the Connectome Workbench^67^.

Registration was performed using the SyN algorithm, a non-rigid transformation in ANTs software^68^.

### Supplementary Information

Supplementary Figure 1.

Supplementary Figure 2.

Supplementary Figure 3.

Supplementary Figure 4.

Supplementary Table 1.

Supplementary Table 2.

## Data Availability

The datasets generated and analyzed during the current study are available in the Brain/MINDS Data Portal (10.24475/bminds.mri.thj.4624).
