# Supplementary material for: Commonality and variance of resting-state networks in common marmoset brains
Source: Sci Rep. 2024 Apr 9;14:8316. doi: 10.1038/s41598-024-58799-w (PMC11004137; doi:10.1038/s41598-024-58799-w)
Supplement: Supplementary file 1 — Supplementary Figure 1. [file 41598_2024_58799_MOESM1_ESM.docx]

**Supplemental Figure 1. TOPUP correction and normalized results in each subject.**

The figure shows the 5 axial sagittal slices of the image for each subject before TOPUP correction (left images), after TOPUP correction (central images), and then normalized to the template image (right images). A-F denotes each individual. The TOPUP correction provided the magnetic resonance images with reduced distortion caused by echo planar imaging.
