# Supplementary material for: Commonality and variance of resting-state networks in common marmoset brains
Source: Sci Rep. 2024 Apr 9;14:8316. doi: 10.1038/s41598-024-58799-w (PMC11004137; doi:10.1038/s41598-024-58799-w)
Supplement: Supplementary file 2 — Supplementary Figure 2. [file 41598_2024_58799_MOESM2_ESM.docx]

**Supplemental Figure 2. TOPUP correction and normalized results in each subject.**

All the images in this figure do not show weak areas with signal intensities below 10. (A) shows the 5 axial slices of the image for each subject before TOPUP correction (left images), after TOPUP correction (central images), and then normalized to the template image (right images). Similar to (A), (B) and (C) show the 5 coronal slices and the 5 sagittal slices, respectively. The TOPUP correction provided the magnetic resonance (MR) images with reduced distortion caused by echo planar imaging (EPI).
