# Supplementary material for: Commonality and variance of resting-state networks in common marmoset brains
Source: Sci Rep. 2024 Apr 9;14:8316. doi: 10.1038/s41598-024-58799-w (PMC11004137; doi:10.1038/s41598-024-58799-w)
Supplement: Supplementary file 3 — Supplementary Figure 3. [file 41598_2024_58799_MOESM3_ESM.docx]

**Supplemental Figure 3. Estimated head motion in each subject and ex vivo brain.**

(A) shows the head motion (translation) before and after motion correction for each session of each subject and in the ex vivo brain. (B) shows the head motion (rotation) before and after motion correction for each session of each subject and in the ex vivo brain. Subject C had a total of 72 sessions of resting-state functional magnetic resonance imaging data, Subjects A and G had a total of 84 sessions, and the other subjects had 120 sessions.
