# Supplementary material for: Commonality and variance of resting-state networks in common marmoset brains
Source: Sci Rep. 2024 Apr 9;14:8316. doi: 10.1038/s41598-024-58799-w (PMC11004137; doi:10.1038/s41598-024-58799-w)
Supplement: Supplementary file 4 — Supplementary Figure 4. [file 41598_2024_58799_MOESM4_ESM.docx]

**Supplemental Figure 4. The flat map of cortical region atlas used in this study and the abbreviations of each region.**

The flat map of the cortical region atlas from the Brain/MINDS Marmoset Reference Atlas (BMA), which was modified from the atlas reported in previous studies (Paxinos G et al., 2012; Hashikawa T et al., 2015; Woodward A et al., 2018). The abbreviation of each region is shown in Supplemental Table 1.
