# Supplementary material for: Commonality and variance of resting-state networks in common marmoset brains
Source: Sci Rep. 2024 Apr 9;14:8316. doi: 10.1038/s41598-024-58799-w (PMC11004137; doi:10.1038/s41598-024-58799-w)
Supplement: Supplementary file 5 — Supplementary Table 1. [file 41598_2024_58799_MOESM5_ESM.docx]

**Supplemental Table 1. The region names and its abbreviation of the cortical region.**

| **Region** | **Abbreviation** |
| --- | --- |
| areas 1 and 2 of cortex | A1/A2 |
| area 10 of cortex | A10 |
| area 11 of cortex | A11 |
| area 13 of cortex lateral part | A13L |
| area 13 of cortex medial part | A13M |
| area 13a of cortex | A13a |
| area 13b of cortex | A13b |
| area 14 of cortex caudal part | A14C |
| area 14 of cortex rostral part | A14R |
| area 19 of cortex dorsointermediate part | A19DI |
| area 19 of cortex medial part | A19M |
| area 23 of cortex ventral part | A23V |
| area 23a of cortex | A23a |
| area 23b of cortex | A23b |
| area 23c of cortex | A23c |
| area 24a of cortex | A24a |
| area 24b of cortex | A24b |
| area 24c of cortex | A24c |
| area 24d of cortex | A24d |
| area 25 of cortex | A25 |
| area 29a-c of cortex | A29a-c |
| area 29d of cortex | A29d |
| area 30 of cortex | A30 |
| area 31 of cortex | A31 |
| area 32 of cortex | A32 |
| area 32 of cortex ventral part | A32V |
| area 35 of cortex | A35 |
| area 36 of cortex | A36 |
| area 3a of cortex (somatosensory) | A3a |
| area 3b of cortex (somatosensory) | A3b |
| area 45 of cortex | A45 |
| area 46 of cortex dorsal part | A46D |
| area 46 of cortex ventral part | A46V |
| area 47 (old 12) of cortex lateral part | A47L |
| area 47 (old 12) of cortex medial part | A47M |
| area 47 (old 12) of cortex orbital part | A47O |
| area 4 of cortex parts a and b (primary motor) | A4ab |
| area 4 of cortex part c (primary motor) | A4c |
| area 6 of cortex dorsocaudal part | A6DC |
| area 6 of cortex dorsorostral part | A6DR |
| area 6 of cortex medial (supplementary motor) part | A6M |
| area 6 of cortex ventral part a | A6Va |
| area 6 of cortex ventral part b | A6Vb |
| area 8 of cortex caudal part | A8C |
| area 8a of cortex dorsal part | A8aD |
| area 8a of cortex ventral par | A8Av |
| area 8b of cortex | A8b |
| area 9 of cortex | A9 |
| agranular insular cortex | AI |
| anterior intraparietal area of cortex | AIP |
| amygdalopiriform transition area | Apri |
| auditory cortex primary area | AuA1 |
| auditory cortex anterolateral area | AuAL |
| auditory cortex caudolateral area | AuCL |
| auditory cortex caudomedial area | AuCM |
| auditory cortex caudal parabelt area | AuCPB |
| auditory cortex middle lateral area | AuML |
| auditory cortex rostral area | AuR |
| auditory cortex rostromedial area | AuRM |
| auditory cortex rostral parabelt | AuRPB |
| auditory cortex rostrotemporal | AuRT |
| auditory cortex rostrotemporal lateral area | AuRTL |
| auditory cortex rostrotemporal medial area | AuRTM |
| dysgranular insular cortex | DI |
| entorhinal cortex | Ent |
| fundus of superior temporal sulcus area of cortex | FST |
| granular insular cortex | GI |
| gustatory cortex | Gu |
| insular proisocortex | Ipro |
| lateral intraparietal area of cortex | LIP |
| medial intraparietal area of cortex | MIP |
| medial superior temporal area of cortex | MST |
| orbital periallocortex | OPAl |
| orbital proisocortex | OPro |
| occipito-parietal transitional area of cortex | OPt |
| parietal area PE | PE |
| parietal area PE caudal part | PEC |
| parietal area PF (cortex) | PF |
| parietal area PFG (cortex) | PFG |
| parietal area PG | PG |
| parietal area PG medial part (cortex) | PGM |
| parietal areas PGa and IPa | PGa - IPa |
| parainsular cortex lateral part | PaIL |
| parainsular cortex medial part | PaIM |
| piriform cortex | Pir |
| proisocortical motor region (precentral opercular cortex) | ProM |
| prostriate area | ProSt |
| retroinsular area (cortex) | ReI |
| secondary somatosensory cortex external part | S2E |
| secondary somatosensory cortex internal part | S2I |
| secondary somatosensory cortex parietal rostral area | S2PR |
| secondary somatosensory cortex parietal ventral area | S2PV |
| superior temporal rostral area (cortex) | STR |
| temporal area TE1 (inferior temporal cortex) | TE1 |
| temporal area TE2 (inferior temporal cortex) | TE2 |
| temporal area TE3 (inferior temporal cortex) | TE3 |
| temporal area TE occipital part | TEO |
| temporal area TF | TF |
| temporal area TF occipital part | TFO |
| temporal area TH | TH |
| temporal area TL | TL |
| temporal area TL occipital part | TLO |
| temporo-parieto-occipital association area | TPO |
| temporopolar proisocortex | TPPro |
| temporal proisocortex | Tpro |
| temporoparietal transitional area | TPt |
| primary visual cortex | V1 |
| visual area 2 | V2 |
| visual area 3 (ventrolateral posterior area) | V3 |
| visual area 3A (dorsoanterior area) | V3A |
| visual area 4 (ventrolatereral anterior area) | V4 |
| visual area 4 transitional part (middle temporal crescent) | V4T |
| visual area 5 (middle temporal area) | V5 |
| visual area 6 (dorsomedial area) | V6 |
| visual area 6A (posterior parietal medial area) | V6A |
| ventral intraparietal area of cortex | VIP |
