# Supplementary material for: Commonality and variance of resting-state networks in common marmoset brains
Source: Sci Rep. 2024 Apr 9;14:8316. doi: 10.1038/s41598-024-58799-w (PMC11004137; doi:10.1038/s41598-024-58799-w)
Supplement: Supplementary file 6 — Supplementary Table 2. [file 41598_2024_58799_MOESM6_ESM.docx]

**Supplemental Table 2. The region names and its abbreviation of the subcortical region.**

| **Region** | **Abbreviation** |
| --- | --- |
| Acumbens | Acb |
| Amygdala | Amy |
| Caudate | Cd |
| Cerebellum | Cer |
| Claustrum and endopirform claustrum | Cl |
| Globus pallidus | GP |
| habenular nuclei | HB |
| Hippocampal formation | HipF |
| Hypothalamus | Hypo |
| Inferior colliculus | IC |
| Lateral geniculate nucleus | LGN |
| Medial geniculate nucleus | MGN |
| Periaqueductal gray | PAG |
| Putamen | Pu |
| Septum | Sep |
| Substantia nigra | SNR |
| Subthalamus | Sth |
| Superior colliculus | SC |
| Thalamus | Thal |
| Zona incerta | ZI |
